# Removal of yttrium from rare-earth wastewater by *Serratia marcescens*: biosorption optimization and mechanisms studies

**DOI:** 10.1038/s41598-022-08542-0

**Published:** 2022-03-22

**Authors:** Chang-li Liang, Ji-li Shen

**Affiliations:** 1 School of Resources and Environmental Engineering, Jiangxi University of Science and Technology, Ganzhou, 341000 China; 2 Huanghuai University, Zhumadian, 463000 China

**Keywords:** Pollution remediation, Microbiology techniques

## Abstract

The discharge of yttrium containing wastewater is a potential risk to human health. Although biosorption is a promising method to remove yttrium from wastewater, whereas the application of it is limited due to the lack of efficient biosorbents. In this study, the removal of yttrium from wastewater using *Serratia marcescens* as a biosorbent was conducted. The effects of six parameters including pH (2–5.5), initial yttrium concentration (10–110 mg/L), biosorbent dosage (0.1–0.5 g/L), biosorption time (10–700 min), stirring speed (50–300 rpm) and temperature (20–60 °C) were evaluated. The main parameters were optimized using response surface methodology. The results showed that the adsorption capacity reached 123.65 mg/g at the optimized conditions. The biosorption mechanism was revealed based on a combined analysis using field emission transmission electron microscope-energy dispersion spectrum, Fourier transform infrared spectrophotometer, and X-ray photoelectron spectroscopy. These results revealed that the hydroxyl, carboxyl, and amino groups were the adsorption functional groups for yttrium ions. Biosorption of yttrium by *S. marcescens* is under the combination of ion exchange, electrostatic attraction and complexation. These findings indicated that *S. marcescens* can be used as an efficient biosorbent to remove yttrium from wastewater. In addition, its adsorption capacity can be further improved by the enhancement of adsorption functional groups on the surface through chemical modification.

## Introduction

Rare Earth Elements (REEs) consists of 15 Lanthanides and two pseudo lanthanides (Scandium and Yttrium), which generally be dividied into Light rare earth elements (LREEs) and heavy rare earth elements (HREEs). REEs play irreplaceable roles in global clean energy technology development, and are termed as the “industrial vitamin”^1,2^. Five critically important REEs (Dy, Eu, Nd, Tb and Y) account for up to ~ 63% of the total weight of all REEs in the final 99.4%-purity product, and yttrium has been widely used in the fields such as catalysts in wide array of industries, sensors, fiber optics and flatten screen display due to its eminent physicochemical properties^3,4^. Consequently, huge amount of yttrium containing wastewater produced along with the mining, industrial processing and purfication is prone to be gradually accumulated in the ecosystem, and finally be transferred and enriched in humans and animals bodies via the food chain^3,5^. Recently, some studies have shown that long-term exposure of human to yttrium can cause human lung embolisms, lung cancer and impedance liver function^6–8^. Thus, recovery of yttrium from wastewater not only can alleviate the ecological and health hazards associated with yttrium polluted to aquatic environment, but also can meet the growing global supply demand of yttrium^9^.

Many conventional methods have been used to recovery REEs resource from wastewater including chemical precipitation^10^, ultra filtration^11^, slovent extraction^12^, electrostatic pseudo liquid membrane^13^, and adsorption^9,14^. However, these conventional methods have some limitations such as less effciency, high operational cost, more maintenance expenditure, high usage and leading to new pollution^15^. Biosorption belongs to adsorption, and refers to the adsorption process is use biomaterial or biopolymer mainly including fungi, bacteria, yeasts, algae, plant-derived materials as adsorbents^16^. Recently, biosorption has been highly recommended because it has advantages such as low cost, variety, efficient, high selectivity, renewability, and sutiful for treating low concentrations wastewater in comparison with tranditional methods^17,18^.

Biosorption is a quite complex process involving one or more elementary mechanisms such as electrostatic attraction, ion exchange, surface complexation and precipitation^18,19^. Ion exchange and surface complexation were considered to be mainly responsible for the adsorption of REEs by *Laminaria ochroleuca* and *Porphyra haitanensis* based on inductively coupled plasma mass spectrometry (ICP-MS) and Fourier transformed infrared spectra (FT-IR) analysis^20^. Electrostatic attraction and precipitation were considered to be the main interactions between europium (Eu) and *Thermus scotoductus* SA-01 based on the analysis of FT-IR and high-resolution X-ray photoelectron spectroscopy (HR-XPS)^21^. Ion exchange was confirmed in the biosorption process of Lanthanum and Cerium by brown marine Alga^22^. The analysis of EDS and XPS confirmed that the Sm(III) was complexed with phosphoryl group on the surface of *Pseudomonas fluorescens* (gram-negative bacteria) and *Bacillus subtilis* (gram-positive bacteria)^23^. The main elementary mechanisms of REEs biosorption processes vary with the variation of biosorbents, REEs speciation and biosorption parameters, revealing the elementary mechanisms is significant for the clarifying of biosorption mechanism.

It is considered that one and more functional groups (such as hydroxyl, carboxyl, amine and phosphoryl) on the biosorbents surface are vital to the biosorption of REEs as they can influence the surface charge state, the light metal ions in ion exchange, and surface complexation binding sites^16,18^. Markai et al.^24^ analyzed the adsorption functional groups of *Bacillus subtilis* for Eu(III) at different pH, and they observed that the carboxylic groups were mainly complexed with Eu(III) at pH 5, while phosphoryl and carboxylic were complexed with Eu(III) when pH exceeded 5. Ngwenya et al.^25^ identified the sorption sites on the bacterial surface for lanthanides based on X-ray absorption spectroscopic (XAS) analysis, and they found that phosphoryl mainly complex with light and mostly middle lanthanides (La to Gd), while some middle and heavy lanthanides (Tb to Yb) complex with carboxylate and phosphoryl. Liu et al.^20^ characterized the adsorption sites of *Laminaria ochroleuca* and *Porphyra haitanensis* for REEs by the combined use of ICP-MS and FT-IR, and they suggested that carboxyl, amino, sulfate, and hydroxyl were the adsorption functional groups. Keshtkar et al.^26^ found that the grafting of sulfur groups to the surface of brown algae *Cystoseira indica* significant enhanced the adsorption capacities for La(III) and Ce(III). Vijayaraghavan and Yun^27^ observed that the decarboxylation of *Corynebacterium glutamicum* made its maximum adsorption capacity for reactive black doubled. Hosomomi et al.^28^ found that the maximum adsorption capacity of *E. coli* modified by diglycolic amic acid for Nd (III), Dy (III) and Lu (III) was 2.63, 2.15 and 1.65 times of raw *E. coli*, respectively. Above studies indicated that the enhancement of the adsorption functional groups on the biosorbents through chemical modification of biosorbents intend to improve the adsorption capacity of biosorbents^29,30^. Therefore, characterization of the adsorption functional groups of biosorbents is the precondition to reveal the biosorption mechanism and to prepare efficient biosorbents for remove REEs from wastewater via biosorption.

Recently, some contributions have focused on the removal of Y(III) ions from wastewater by biosorption to eliminate the risks to the human health. Pinto et al.^31^ evaluated that the biosorption of Y(III) ions by six living microalgae. They found that the adsorption capacity of the six living microalgae ranged from 0.2 mg/g to 1.05 mg/g, respectively. Karavaiko et al.^32^ studied the selectivity recovery scandium and yttrium ions from the acid solution by *Saccharomyces cerevisiae* and *Aspergillus terreus* via biosorption. They found that the maximum adsorption capacity of *Saccharomyces cerevisiae* and *Aspergillus terreus* for Y(III) was 40 mg/g and 15 mg/g at pH 4.5, respectively. Hussien and Desouky^33^ reported the maximum adsorption capacity of *Pleurotus Ostreatus* preteated by NaOH for Y(III) reached 45.45 mg/g. Overally, the reported adsorption capacity of microorganisms for yttrium is unsatisfied, and the biosorption mechanism for yttrium is still unclear. It is necessary to screen out the efficient biosorbents for yttrium and to further elucidate the adsorption mechanism.

In this work, *Serratia marcescens* (*S. marcescens*) isolated from the wastewater of a heavy yttrium rare-earth mining area in the Jiangxi Province (China), and it was used as the biosorbent to remove Y(III) from wastewater by biosorption. The aim of this work was to optimize the biosorption parameters and reveal the biosorption mechanism of *S. marcescens* for Y(III). Single-factor experiments were performed to determine the main influence factors. Further optimization was done based on the selected main biosorption parameters using response surface methodology (RSM). The elementary mechanisms of the biosorption and the adsorption functional groups were characterized by the combination using field emission transmission electron microscope (FETEM), SEM–EDS, FT-IR and XPS. The results of this study can provide theoretical and technological guidance for the design of efficient biosorbents for Y(III) removal from wastewater via biosorption.

## Materials and methods

### Preparation of *S. marcescens*

*Serratia marcescens* was isolated from the wastewater of a heavy yttrium rare-earth mining area in Jiangxi Province (China), and it was domesticated in Y(III) solution. Monoclones were picked up and activated on beef extract peptone plates, and then were transferred to a liquid beef extract peptone medium. The cells were cultured in a rotary shaker (150 rpm) at 30 °C for two days. Cells were then harvested during the logarithmic phase by centrifugation (8000 rpm) at 4 °C for 20 min, after which the cells were washed three times using deionized water. The biomass was then freezing-dried as biosorbents.

### Synthetic yttrium wastewater

All the chemical reagents used in the study were analytical grade and purchased from Bioengineering, Shanghai, China. A synthetic yttrium stock solution (1 g/L) was prepared by dissolving 1 g Y_2_O_3_ (99.99%, Bioengineering, Shanghai, China) in 300 mL deionized water and adding the appropriate volume of 0.1 M H_2_SO_4_. The solution was heated and stirred until completely dissolved. Once the solution was cooled to room temperature, deionized water was added till 1 L. The concentration of Y(III) stock solution was analyzed by ICP-OES (CAP7000, Thermo Scientific, America). Stock Y(III) solutions were stored at 4 °C.

### Biosorption experiments

Biosorption tests were conducted in 150 mL Erlenmeyer flask contained 100 mL Y(III) solution and stirred adsorption in a constant-temperature oscillator for a certain time by the batch technique. Single-factor experiments were conducted at sequent biosorption parameters including, solution pH (2–5.5), Y(III) concentration (10–110 mg/L), biosorbent dosage (0.1–0.5 g/L), biosorption time (10–700 min), stirring speed (50–300 rpm) and temperature (20–60 °C). When the adsorption reached equilibrium, the solution was centrifuged (12,000 rpm for 15 min) at 4 °C. Y(III) concentration of the supernatant was detected with an ICP-OES. Results presented in this study are mean values obtained from triplicate analysis under identical conditions. The removal rate and adsorption capacity of the biosorbent were calculated according to Eqs. (1) and (2), respectively.

1 $${\text{Adsorption capacity}} = \left( {C_{0} - C_{e} } \right) \times \frac{V}{m}$$

2 $${\text{Removal rate}} = \frac{{C_{0} - C_{e} }}{{C_{0} }}*100\%$$

where C_0_ and C_e_ represent the initial and equilibrium concentrations of Y(III) (mg/L), respectively; m is the mass of the adsorbent (g); and V is the volume of the solution (L).

The biosorption parameters of *S. marcescens* for Y(III) were optimized by RSM. Biosorbent dosage, pH, initial Y (III) concentration and stirring speed were selected as the design variables based on the results of single-factor experiments. The coding limits and levels of these variables are shown in Table 1. Twenty-nine tests were designed by using Box-Behnken design (BBD). Moreover, the biosorption time and temperature was constant to 240 min and 25 °C for all the optimization tests. RSM data and the validity of the proposed model were analyzed using design expert 8.06(Stat Ease Inc., UK).

**Table 1:** Design of the coding limits and levels of independent variables.

Independent variable	Variables	Variables level		
		− 1	0	+ 1
pH	A	4.5	5	5.5
Biosorbent dosage (g/L)	B	0.3	0.4	0.5
Initial Y(III) (mg/L)	C	30	50	70
Stirring speed (rpm)	D	150	200	250

### Characterization

*Serratia marcescens* samples were prepared by adding 0.11 g *S. marcescens* into the 500 mL Erlenmeyer flask filled with 300 mL 56.41 mg/L Y(III) solution (pH 5.5). The samples were kept in a constant-temperature in an oscillator (25 °C, 169 rpm) biosorption for 240 min, and three tests were performed. When the adsorption reached equilibrium, the solution was centrifuged (12,000 rpm for 15 min) at 4 °C. The *S. marcescens* cells were washed with deionized water, and were then freezing-dried for characterization.

The *S. marcescens* samples analyzed by FETEM were mainly prepared as the following procedure: *S. marcescens* before and after Y(III) biosorption were fixed with glutaraldehyde, then they were washed with cacodylate. Afterwards, the samples were dehydrated with graded ethanol and then resin embedded. Samples were analyzed using FETEM (FEI Tecnai G2F30, FEI Company, American) equipped EDS*.*

The surface morphology of *S. marcescens* before and after Y(III) biosorption was recorded by MLA650F(FEI, America). The cell surface elemental composition was detected by EDS (Bruker, Germany). Infrared spectra of *S. marcescens* before and after Y (III) biosorption were recorded in the range of 4000–800 cm^−1^ using a Nicolet is 5 Fourier transform infrared spectrophotometer (Thermo Scientific, America). High-resolution C1s, N1s, O1s spectra of *S. marcescens* before and after Y(III) biosorption were recorded using a Thermo Escalab 250XI X-ray photoelectron spectrometer (Thermo Scientific, America). The spectra were fitted and analyzed using Advantage (Thermo Scientific, America).

### Ethics approval and consent to participate

This article does not contain any studies with human participants or animals performed by any of the authors.

### Consent to participate

All authors agree to participate.

### Consent to publish

All authors consent to publish.

## Results and discussion

### Single-factor experiment

**Figure 1:**
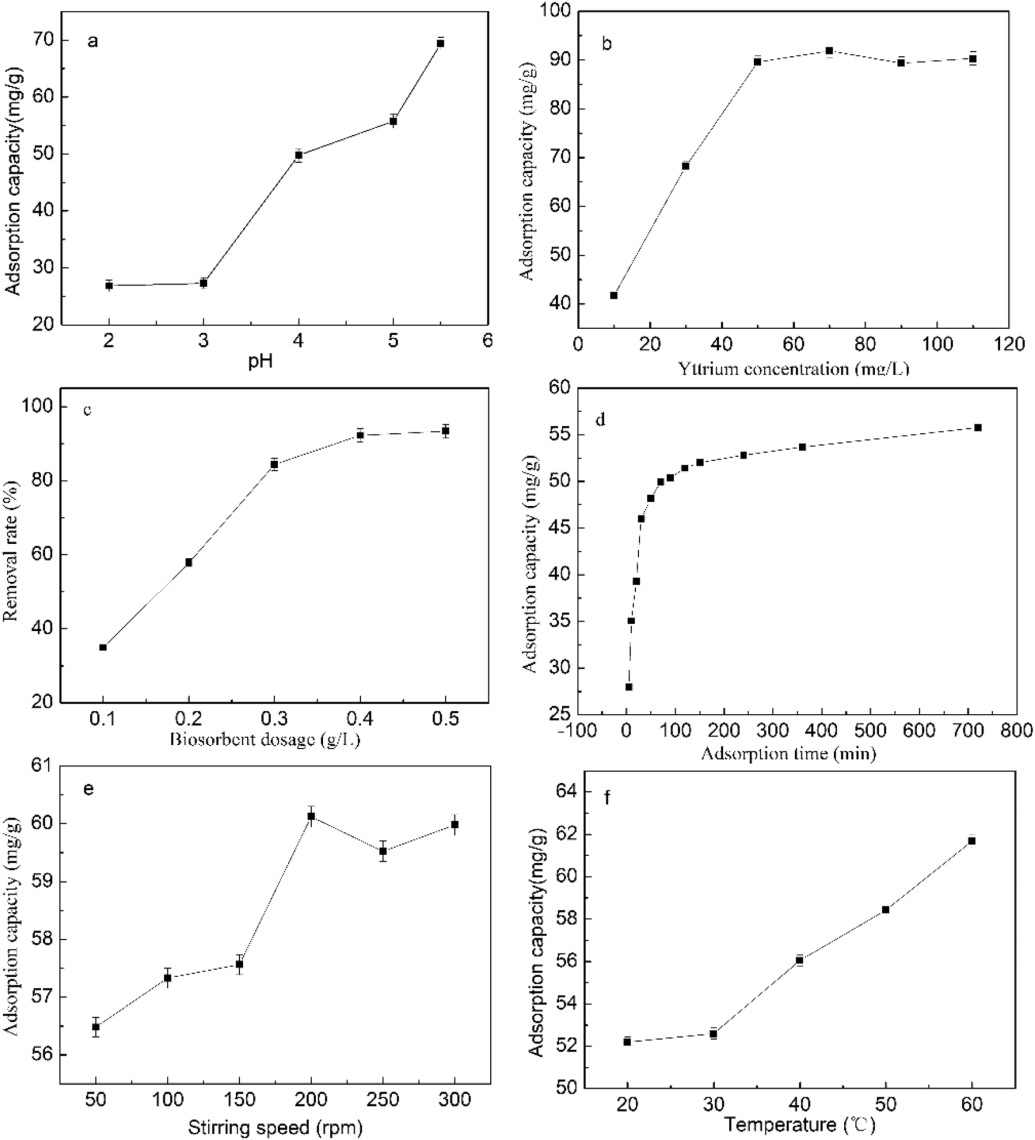
Effect of pH (**a**), initial Y(III) concentration (**b**), biosorbent dosage (**c**), adsorption time (**d**), stirring speed (**e**) and temperature (**f**) on the adsorption capacity of *S. marcescens* for Y(III) (The initial adsorption quantitative conditions were set as pH ranged from 2 to 5.5, initial Y(III) concentration of 50 mg/L, biosorbent dosage of 0.3 g/L, stirring speed of 150 rpm, biosorption of 25 °C, solution volume of 100 mL and biosorption for 60 min).

The effect of biosorption parameters (pH, biosorbent dosage, Y(III) concentration, adsorption time, temperature, and stirring speed) on the adsorption capacity of *S. marcescens* for Y(III) are shown in (Fig. 1a–f).

The pH of the biosorption solution not only influences the surface charge of biosorbents, but influences the chemical state of metal ions. As shown in Fig. 1a, the biosorption process can be divided into a slow biosorption stage (pH 2–3) and a rapid biosorption stage (pH 3–5.5). Adsorption capacity was lower than 30 mg/g in the pH interval of 2.0–3.0, as the surface of *S. marcescens* was positively charged when the solution pH was lower than its isoelectric point (4.47, data not shown) which led to electrostatic repulsion. Wang and Wang^34^ also reported a rapid decrease in the adsorption capacity caused by the protonation of carboxyl groups when the pH was lower than 3.0. In addition, the adsorption capacity rapidly increased from 27.3 to 69.4 mg/g with the increase of pH from 3.0 to 5.5. In fact, this rapid increased of adsorption capacity was due to the increase of the surface electronegativity, which favored the Y(III) adsorption onto *S. marcescens* through electrostatic attraction. Kazak et al.^35^ also observed that the adsorption capacity of five heterotrophic bacteria strains to REEs at pH 4 was all higher than those recorded at pH 2. Therefore, the results of the present study confirmed the occurrence of electrostatic attraction during biosorption. The maximum pH value of 5.5 was studied in the current study because the Y(III) ion precipitate was observed when the solution pH exceeded 5.5, which can influence the biosorption assessment. Therefore, a pH value of 5.5 was used in the following single-factor experiments.

Figure 1b shows that the adsorption capacity quickly increased with the increase of Y(III) concentration when it was lower than 70 mg/L, and the adsorption capacity recorded a decreasing, oscillating trend when Y(III) concentration exceeded 70 mg/L A maximum adsorption capacity of 91.88 mg/L was achieved at 70 mg/L. This result indicated that the unoccupied adsorption sites of biosorbent decreased with an increase in initial Y(III) concentration, and gradually became saturated when Y(III) concentrations exceeded 70 mg/L^36^. Therefore, 70 mg/L Y(III) solution was used in the following experiments.

As shown in Fig. 1c, the removal rate increased with the increase of biosorbent dosage and reached 92.25% at 0.4 g/L. Obviously, the biosorption sites increased with the increase of biosorbents and enhanced the biosorption of Y(III). The biosorption reached equilibrium when the biosorbent dosage exceeded 0.4 g/L. This may be due to the significant decrease of free Y(III) in the bulk solution and the biosorption thus reached saturation^37^. Therefore, the biosorbent dosage of 0.4 g/L was used in the following single-factor experiments.

As shown in Fig. 1d, biosorption mainly occurred at the initial 90 min, and an increase from 28 to 50 mg/g was recorded in the time interval. When adsorption time increased from 240 to 720 min, adsorption capacity slowly increased from 52.82 to 55.78 mg/g. Rapid adsorption was mainly because the majority of biosorption sites were unoccupied in the initial stage, and adsorption became more difficult due to the fact that most adsorption sites were occupied and the biosorption became saturation^38^. Therefore, to ensure the biosorption reach equilibrium, biosorption for 240 min in the following single-factor experiments was applied.

Adsorption capacity increased with the increase of stirring speed from 50 to 200 rpm, and the adsorption capacity fluctuated when the stirring speed exceed 200 rpm (Fig. 1e). This result is consistent with the study by Hussien and Desouky^33^, and they considered high stirring speed favor of metal ions diffuse from bulk solution to the surface of biosorbents. Therefore, the stirring speed of 200 rpm was used in the following single-factor experiments.

As biosorption temperature increased from 20 to 60 °C, adsorption capacity increased from 52.19 to 61.69 mg/g (Fig. 1f). This result showed that the biosorption is an endothermic process, which is consistent with the findings of Hussin and Desouky^33^. Although the adsorption capacity increase 18% when temperature increased from 20 to 60 °C, the increase of biosorption temperature will result in the uneconomic of the biosorption process for it required more energy. Therefore, the biosorption temperature was not regarded as the main parameters for the optimization.

The results of the single-factor experiments showed that the solution pH, biosorbent dosage, initial yttrium concentration and stirring speed had important influence on Y(III) ions biosorption by *S. marcescens*. Therefore, these factors were taken as variables for optimization of biosorption parameters by RSM.

### Optimization of biosorption parameters by RSM

As shown in Table 2, *p*-values of the factors A (< 0.0001), C (< 0.0001), B (0.0002) and D (0.0002) all lower than 0.05, indicating that these variables significant influenced the biosorption of Y(III) by *S. marcescens*. The *p*-values of interaction terms AB (< 0.0001), AC (0.0172) and BD (0.0078) were all lower than 0.05, indicating that these interaction terms have significantly influence on the biosorption. A second-order polynomial model was recommended by the fitting of the experiments data by design expert software (8.06), and expressed as Eq. (3).

3 $$\begin{aligned} {\text{Adsorption}}\,{\text{capacity}}\,\left( {{\text{mg}}/{\text{g}}} \right) & = {81}.{14} + {2}0.{\text{31 A}} - {9}.0{\text{8 B}} + {18}.{9}0{\text{C}} - {8}.{\text{87 D}} - {18}.{\text{27AB}} \\ & \quad + {8}.{8}0{\text{AC}} - {5}.{\text{99AD}} + {1}.{\text{18BC}} + {1}0.0{\text{5BD}} + {4}.{\text{71CD}} \\ \end{aligned}$$

**Table 2:** Analysis of variance (ANOVA), regression coefficient estimate and test of significance for adsorption capacity of *S. marcescens* for Y (III) by design expert 8.0.6

Source	Sum of squares	*df*	Mean square	F value	p-value Prob > F	
Model	13,454.41	10	1345.44	29.88	< 0.0001	Significant
A-pH	4949.95	1	4949.95	109.94	< 0.0001	
B-Adsorption concentration	988.99	1	988.99	21.97	0.0002	
C-Yttrium concentration	4284.63	1	4284.63	95.17	< 0.0001	
D-Rotate speed	944.66	1	944.66	20.98	0.0002	
AB	1335.17	1	1335.17	29.66	< 0.0001	
AC	309.76	1	309.76	6.88	0.0172	
AD	143.28	1	143.28	3.18	0.0913	
BC	5.52	1	5.52	0.12	0.7302	
BD	403.61	1	403.61	8.96	0.0078	
CD	88.83	1	88.83	1.97	0.1771	
Residual	810.41	18	45.02			
Lack of fit	767.95	14	54.85	5.17	0.0622	Not significant
Pure error	42.46	4	10.61			
Cor total	14,264.81	28				

R^2^: 0.9432; Adjusted R^2^: 0.9116; Adequate precision: 19.477; Predicted R^2^:0.8359.

**Figure 2:**
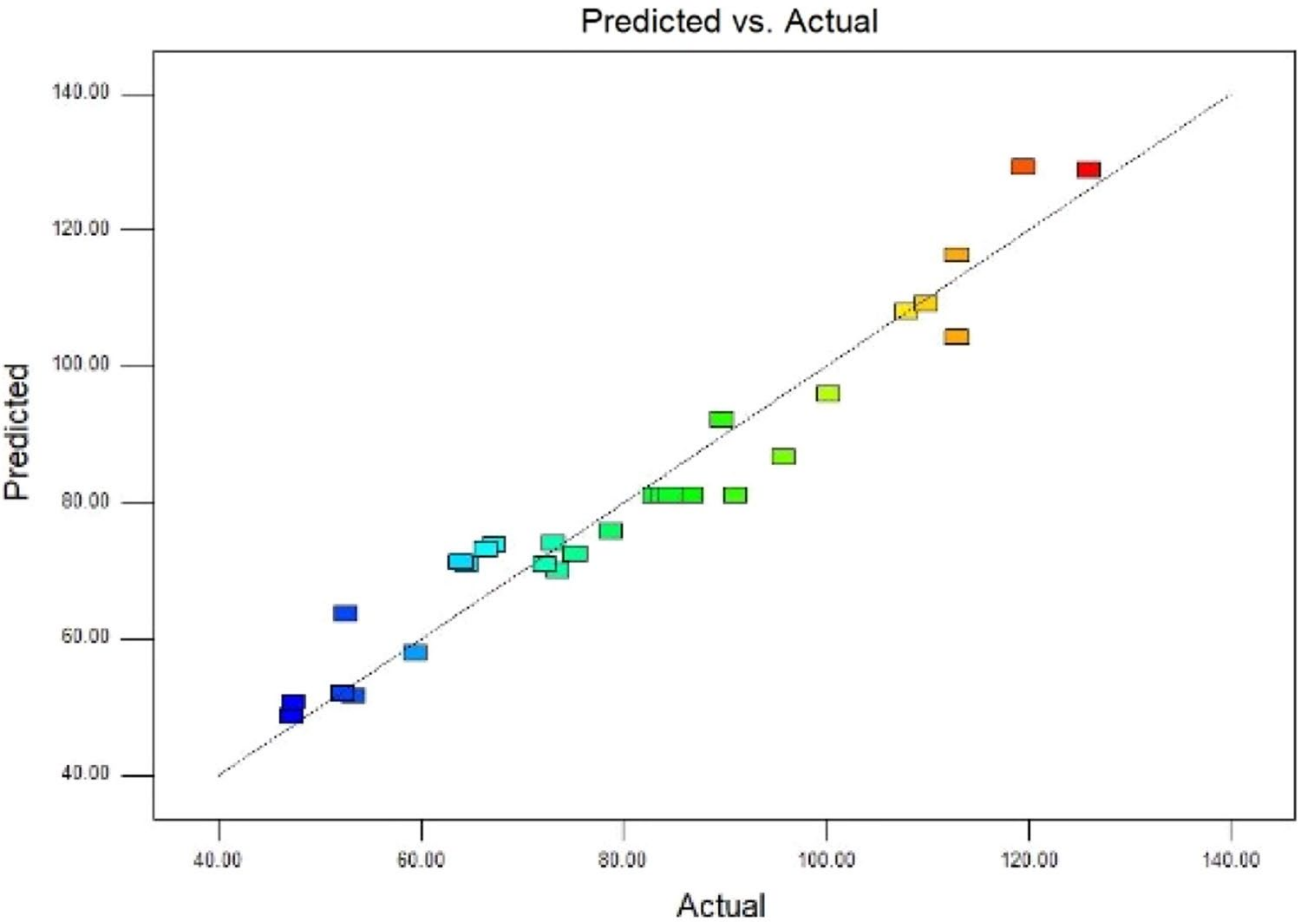
Experiments data vs predicted values for adsorption capacity fitted by design expert 8.06.

The *p* value of the model was < 0.0001 and a high F value of 1345.44 was recorded, confirming that the quadratic model was significant and suitable for the prediction of adsorption capacity of *S. marcescens* for Y(III). Lack of fit (LOF), representing the difference between the model function and the real function, the model is considered to be significant when it is > 0.05^14^. LOF value 0.0622 > 0.05 indicated the conformity of the model (Table 2). A closer the correlation coefficient (R^2^) to 1 indicated a stronger and better model prediction response^39^. Here, R^2^, adjusted R^2^ and predicted R^2^ were 0.9432, 0.9116 and 0.8359, respectively. The results confirmed that a reasonable agreement and that regression was significant. Results presented in Fig. 2 also confirmed that a high level of consistency exists between RSM-based predicted results and experimental data. Adequate precision, an indicator to measure the ratio of signal to noise, was recorded to be 19.477. As this in the current study was substantially higher than 4, which confirmed the validity of the model.

### The effect of variables on the adsorption capacity of *S. marcescens* for Y (III)

**Figure 3:**
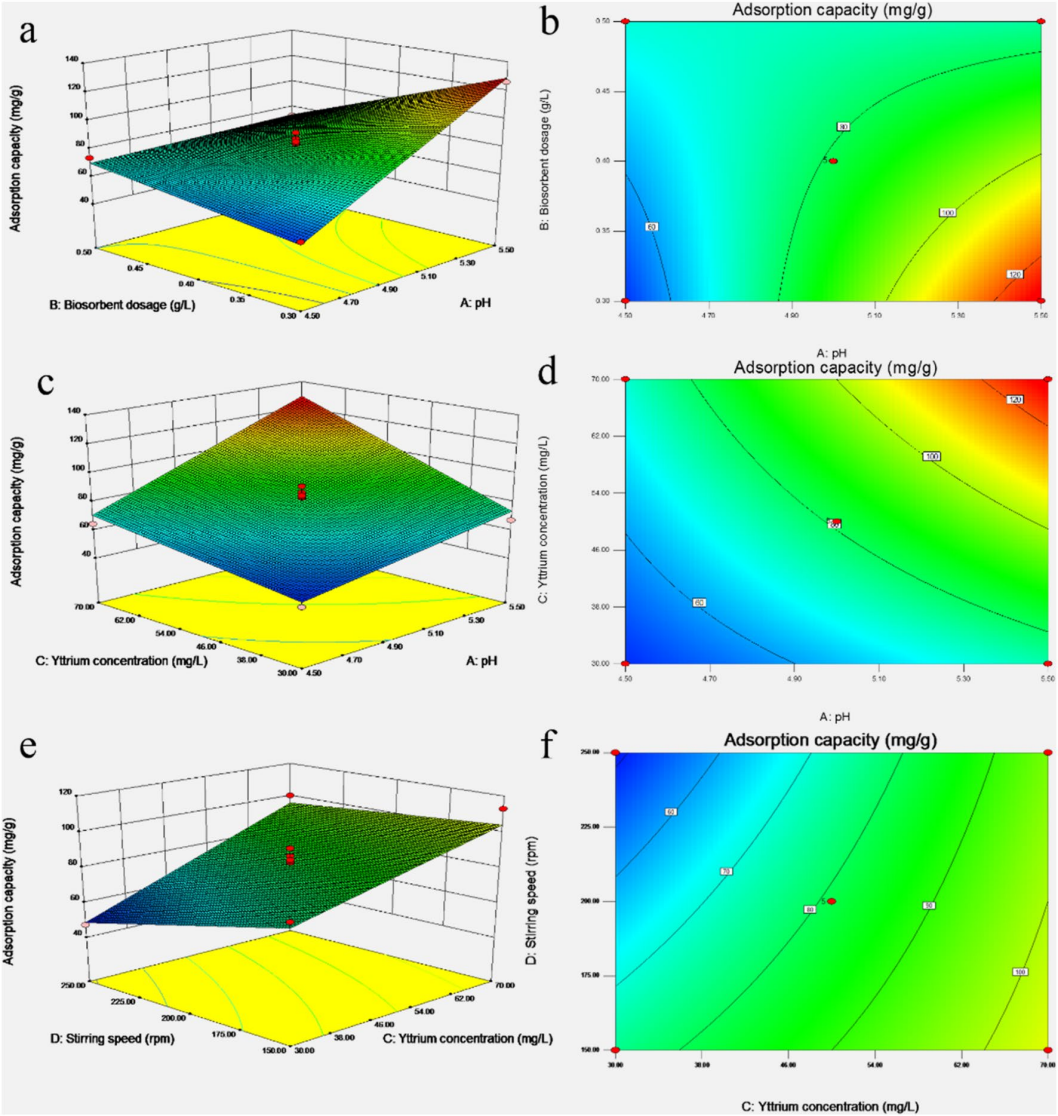
3D surface and contour of adsorption capacity (**a**,**b**) effect of pH and biosorbent dosage, (**b**,**c**) effect of pH and Y(III) concentration, (**e**,**f**) effect of Y(III) and stirring speed (analyzed by design expert 8.06).

The interactions among the variables were evaluated using 3D response surface and contour plots. The 3D curves of the interaction effects of pH and biosorbent dosage, pH and initial Y(III) concentration, initial Y(III) concentration and stirring speed on the adsorption capacity are shown in Fig. 3a–f, respectively.

The pH of the biosorption solution is a significant factor influences the adsorption capacity of biosorbents by influencing the charged conditions of the biosorbents and the ionization species of biosorbents^35^. As pH increased in the current experiments, the adsorption capacity also increased (Fig. 3a,c). However, the increase of biosorbent dosage decreased the adsorption capacity (Fig. 3a). Mauricio et al.^40^ reported that the maximum adsorption capacity of *Sargassum fluitans* for lanthanum increased more than tenfold as the pH increased from pH 2 to 5. Furthermore, the influence of biosorbent dosage on the adsorption capacity is not significant as pH value. The contour plot (Fig. 3b) confirmed that the results the 3D surface plot were consistent with that of the single-experiment of pH and biosorbent dosage. Moreover, the highest adsorption capacity was observed at high pH levels and low biosorbent dosages. However, the contour plot indicated that the interactions between pH and biosorbent dosages were not significant because the plot is not ellipse^41^.

The effect of initial Y(III) concentration on the adsorption capacity was similar with that of pH values (Fig. 3e,f). Figure 3d showed that the interactions between initial Y(III) concentration and pH was also insignificant. The effect of stirring speed was weak in comparation with that of initial Y(III) concentration, and adsorption capacity decreased as stirring speed increased (Fig. 3e). Higher adsorption capacities obtained at higher initial Y(III) concentrations and lower stirring speed. The contour plot (Fig. 3f) illustrates that there were no strong interactions between initial Y (III) concentration and stirring speed. The result of contour plot showed that the interactions of the studied factors has no significant influence on the adsorption capacity.

The optimal values of the biosorption parameters suggested by the second-order polynomial model were as follows, initial pH (5.35), biosorbent dosage (0.33 g/L), initial Y(III) concentration (56.41 mg/L), stirring speed (169 rpm), 25  °C biosorption for 240 min, and the predicted adsorption capacity was 129.98 mg/g. The adsorption capacity of the verified tests had a mean adsorption capacity of 123.65 mg/g confirming that the experimental results were consistent with the predicted result. Therefore, these results confirmed the validity of the proposed model, and that it can be used in the prediction of the adsorption capacity of *S. marcescens* for Y(III).

Table 3 shows a comparison of the adsorption capacities of different adsorbents for Y(III). The adsorption capacity of *S. marcescens* for Y(III) is obvious higher than the fungi and yeasts^39^ and *Pleurotus ostreatus* pretreated with NaOH^33^, and higher than calcium alginates and near to sodium alginates^42^. Therefore, the results of the present study indicated that the *S. marcescens* was an efficient biosorbent for the removal of yttrium from wastewater via biosorption.

**Table 3:** Comparation of the maximum adsorption capacity of biosorbents for Y(III) in the literatures and the current study.

Adsorbent	Adsorption conditions	Maximum adsorption capacity (mg/g)	References
Fungi	pH: 4.5	35–40	^39^
Yeasts		12–15	
*Pleurotus ostreatus* pretreated with NaOH	50 mg/L Y(III), 150 mg/L dry wt. cell, pH 6.5, 30 °C, 175 rpm, biosorption 2 h	35.27	^33^
Calcium alginates	pH 4.0, stirring speed 200 rpm, 500 mg/L Y(III)	97.79	^42^
Sodium alginates	pH 6.0, stirring speed 200 rpm, 500 mg/L Y(III)	126.3	
*Serratia marcescens*	pH 5.5, stirring speed 169 rpm, 56.41 mg/L, biosorbent dosage is 0.33 g/L	123.65	This paper

### Characterization adsorption sites by FETEM

Biosorbents can adsorb heavy metals on their surface, and biosorbents can bioaccumulate heavy metals inside their cell by metabolic process. Therefore, cell slice morphology and elemental composition inside and outside of *S. marcescens* cells before and after biosorption was characterized by FETEM equipped with EDS to verify the adsorption sites of *S. marcescens* for Y(III).

**Figure 4:**
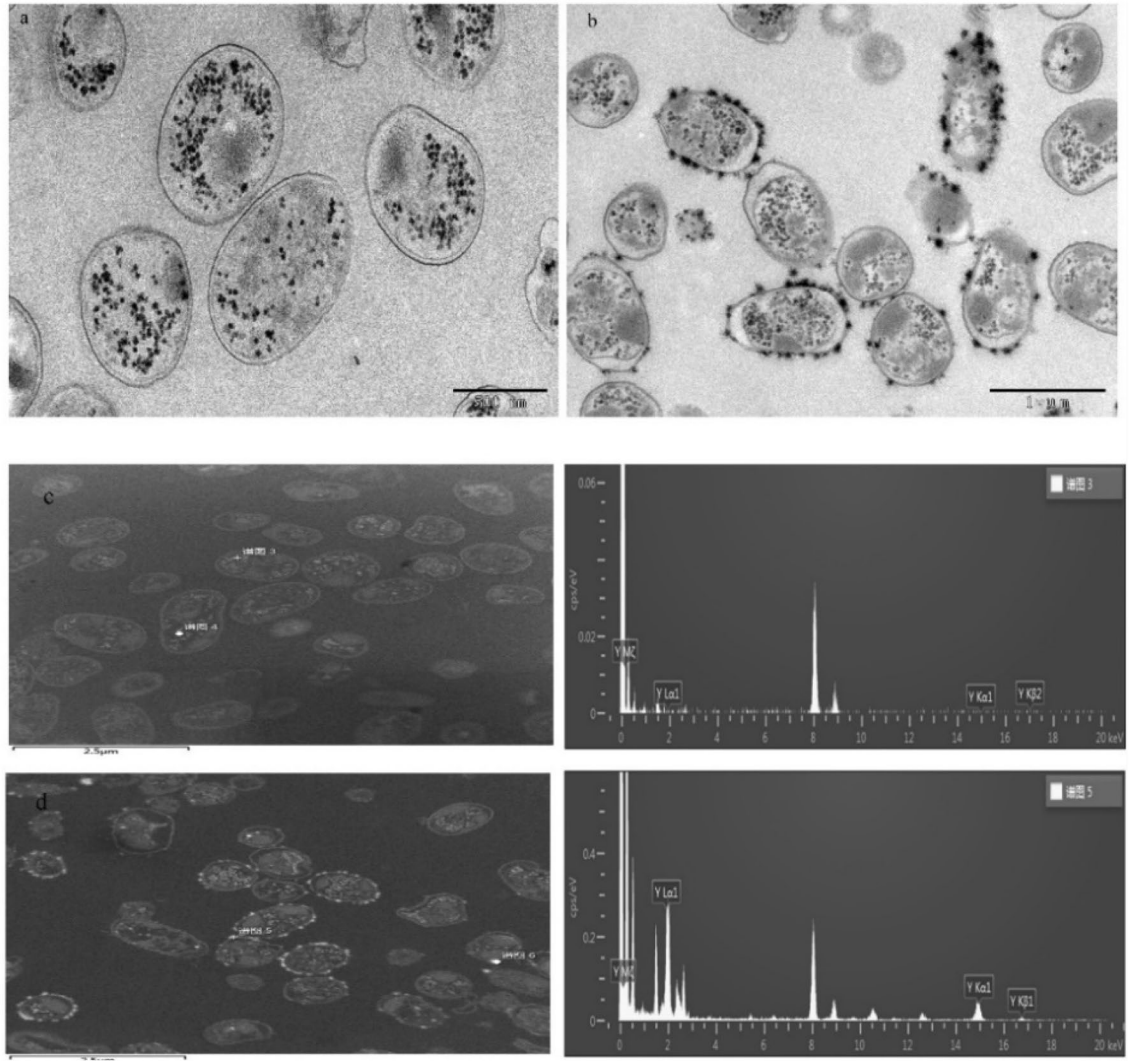
FITEM micrographs of *S. marcescens* before (**a**) and after biosorption Y(III) (**b**), EDS analysis of *S. marcescens* before (**c**) and after biosorption Y(III) (**d**).

The cell wall of raw *S. marcescens* contained some inclusion particles inside the cell (Fig. 4a). Figure 4c illustrates that Y(III) was not present on the cell surface or inside cells of *S. marcescens*. Moreover, differences were observed between micrographs of *S. marcescens* cells before and after biosorption (Fig. 4a,b), and some amorphous substances were observed outside of the cell walls. The analysis of EDS confirmed that amorphous substances outside the cell wall were yttrium (Fig. 4d). These results confirmed that the Y(III) was adsorbed onto the cell surface, which is consistent with previous investigation^20^.

### SEM–EDS analysis

**Figure 5:**
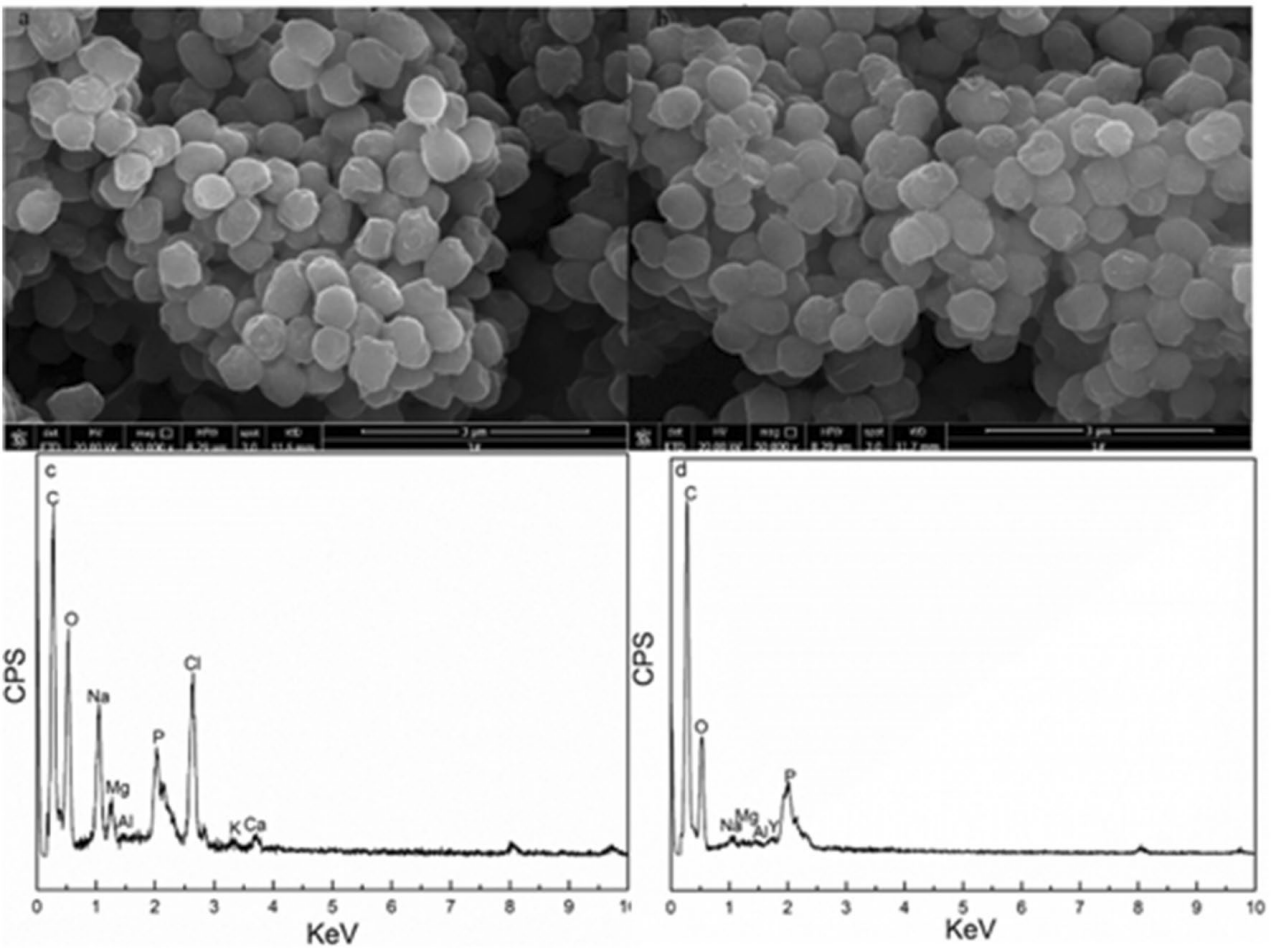
SEM spectra of *S. marcescens* (**a**) before and (**b**) after Y(III) biosorption, EDS spectra of *S. marcescens* (**c**) before and (**d**) after Y(III) biosorption.

Ion exchange is one elementary mechanism of adsorption. Surface morphology and cell surface elemental content were analyzed using SEM-EDS to verify whether the ion exchange action occurs during *S. marcescens* biosorption of Y(III) (Fig. 5 and Table 4).

**Table 4:** EDS analysis of *S. marcescens* surface metallic element content before and after Y(III) biosorption.

Samples	Yttrium (%)	K (%)	Na (%)	Ca (%)	Mg (%)
Before adsorption	0	0.24	2.67	0.09	0.19
After adsorption	0.77	0.02	0.18	0.04	0.11

Comparison of the cell surface of *S. marcescens* before and after biosorption of Y(III) (Fig. 5a,b). Except some deposits on the cell surface of *S. marcescens* after biosorption, no distinct differences were visible on the cell surface after adsorption. The EDS analysis indicated that the intensity of metals on the cell surface all decreased after interaction with Y(III) (Fig. 5c,d). Yttrium content on the surface of *S. marcescens* increased from 0% to 0.77% after biosorption, which confirmed that the yttrium was adsorbed on the cells. Apart from a variation in yttrium content, the content of Ca^2+^, Mg^2+^, Na^+^ and K^+^ all decreased after biosorption, especially Na^+^, Mg^2+^ and K^+^. This result confirmed that the occurrence of ion exchange during adsorption, and ion exchange mainly occurred between Y(III) and Na^+^, Mg^2+^ and K^+^. Liu et al.^20^ also reported that the occurred of ion exchange during REEs ions biosorption on algae.

### FTIR characterization

Some groups of biosorbents play vital roles in the biosorption of heavy metals from an aqueous solution. Therefore, the *S. marcescens* cells before and after biosorption were characterized by FT-IR to reveal the adsorption functional groups of *S. marcescens* for Y(III).

**Figure 6:**
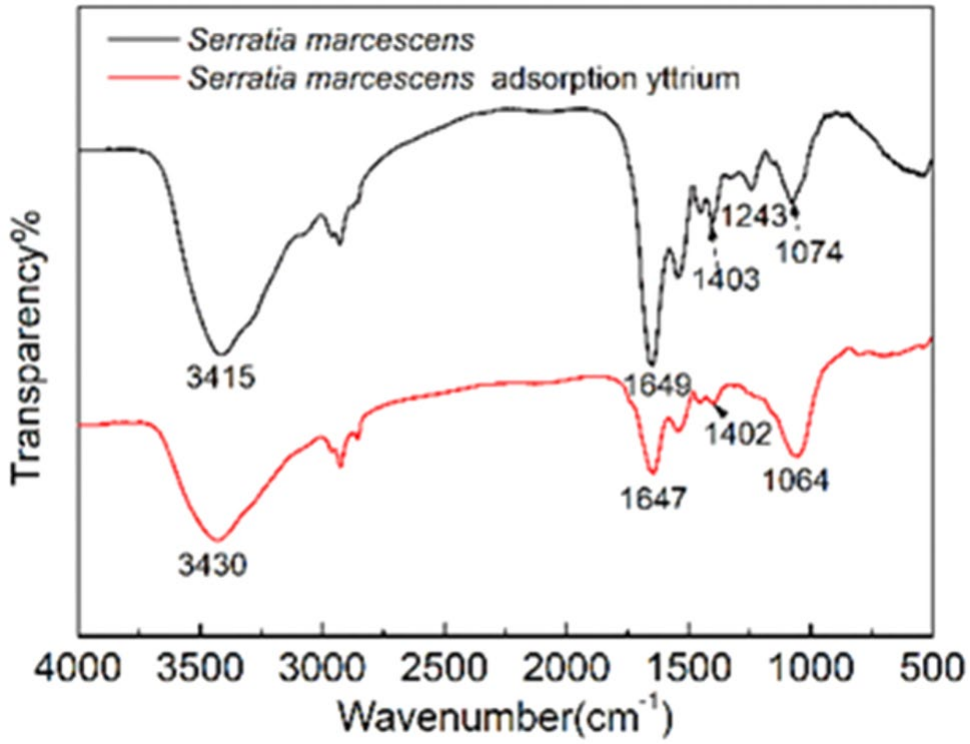
FT-IR spetra of *S. marcescens* before and after Y(III) biosorption.

As shown in Fig. 6, a peak appeared at 3415 cm^−1^ before the biosorption of Y(III). This peak overlapped stretching vibrations in the hydroxyl (–OH) and amine (–NH) groups derived from sugars and amino acids^43,44^. The distinguished asymmetric and symmetric stretching vibration peaks of carboxylate (–COO^−^) were evidenced at 1649 and 1403 cm^−1^^45^. In addition, the peaks recorded at 1243 cm^−1^ and 1074 cm^−1^ were the characteristic peaks of carboxyl/carboxylate and stretching vibrations of C–OH, respectively^46^.

A positive peak shift from 3415 to 3430 cm^−1^ after biosorption should result from the weakening of hydrogen-bonding interactions of O–H or the variation of N–H under the influence of Y(III). The negative shift of the asymmetric stretching vibrations of –COO^−^ to 1643 cm^−1^, and the reduction of the symmetric vibration peak intensity of –COO^−^ were evidenced in the spectra of after the biosorption. These results indicated the formation of polydentate complexes from carboxylate groups covalently bonded with transition metal yttrium^47^. In addition, the diminished peak at 1243 cm^−1^ indicated the involvement of carboxyl groups during biosorption. The negative shift of the peak from 1074 to 1064 cm^−1^ also indicated the involvement of C–OH. The result agrees with Roozegar and Behnam^45^, and they also found a negative shift of C–OH peak after alage biosorption of copper. The analysis of FTIR showed that the hydroxyl, amine, and carboxyl groups derived from proteins and polysaccharides might be involved in the *S. marcescens* biosorption of Y(III).

### XPS characterization

**Figure 7:**
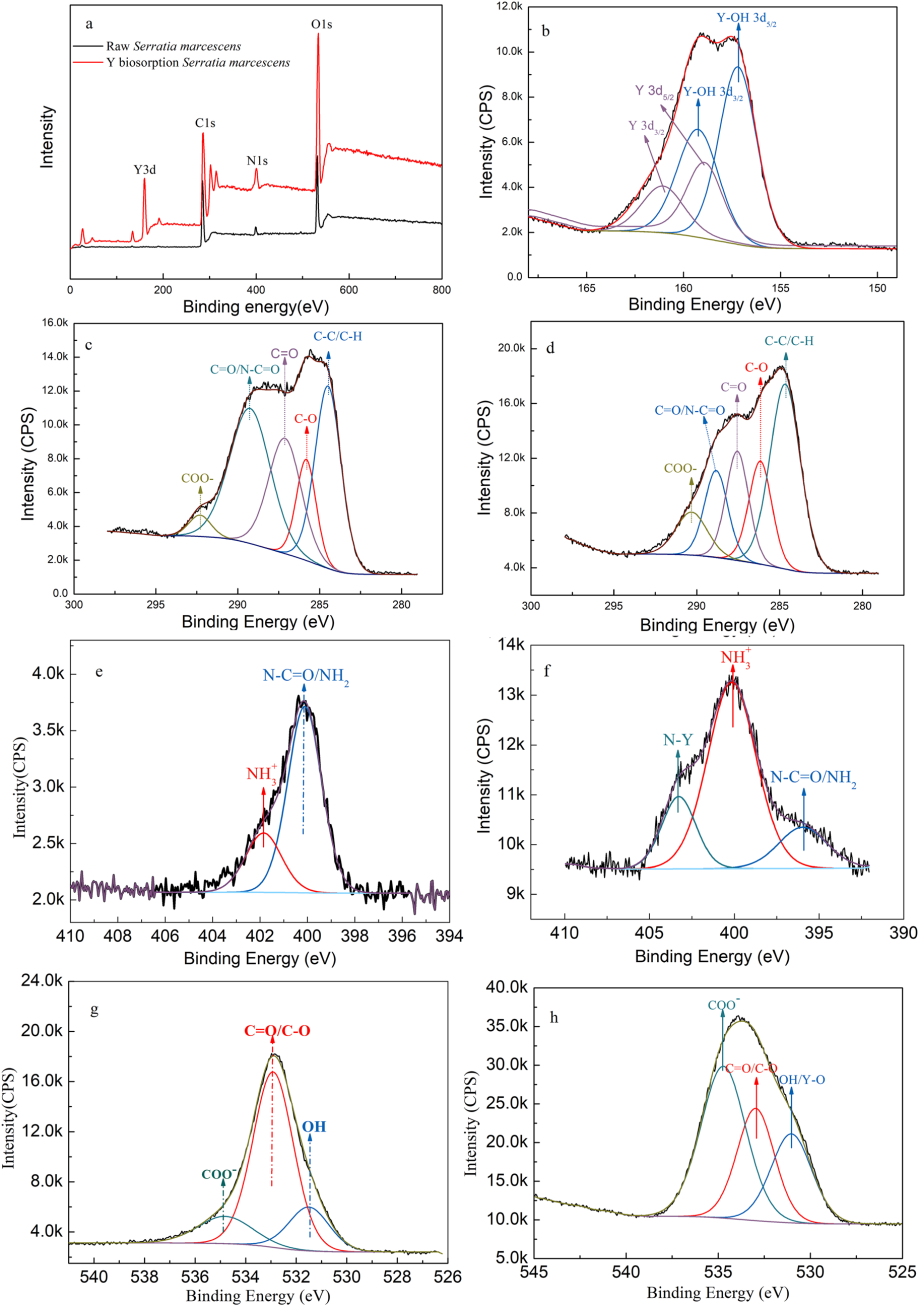
XPS spectra of *S. marcescens*. Survey spectra of *S. marcescens* before and after Y(III) biosorption (**a**), Y3d spectra of *S. marcescens* after Y(III) biosorption (**b**), C1s spectra of *S. marcescens* before (**c**) and after Y(III) biosorption (**d**), N1s spectra of *S. marcescens* before (**e**) and after Y(III) biosorption (**f**), O1s spectra of of *S. marcescens* before (**g**) and after Y(III) biosorption (**h**).

The survey XPS, high-resolution spectra of Y3d, C1s, N1s and O1s spectra for *S. marcescens* before and after biosorption of Y(III) are shown in Fig. 7a–h, respectively. The assignment, binding energy (BE), and the atomic concentration (AC/%) of the components are shown in Table 5.

**Table 5:** Composition in elements (C, N, O, Y) identified, binding energy (BE) and AC (%) of the deconvolution components in the XPS spectra for *S. marcescens* before and after Y(III) biosorption.

Element	Chemical group	*S. marcescens*		*S. marcescens* biosorption Y(III)	
		BE (eV)	AC%	BE (eV)	AC%
C1s	C–C/C–H	284.5	28.82	284.7	40.68
	C–O/C–N	285.8	12.06	286.2	17.14
	C=O/N–C=O	287.1	22.95	288.8	17.80
	C=O/C = N	289.3	24.34	290.1	14.65
	COO-	292.3	11.83	290.31	9.72
N1s	N–C=O/NH_2_	399.8	72.3	396	14.74
	NH_3_^+^	401.6	27.7	400.1	66.99
	N–Y			403.3	18.28
O1s	O–H/Y–O	531.5	17.09	531	24.80
	C=O/C–O	532.9	68.6	533	28.58
	COO^−^	534.82	14.31	534.7	46.62
Y3d	Y-OH 3d5/2			157.23	72.85
	Y-OH 3d3/2			159.28	
	Y3d3/2			161.08	27.15
	Y3d5/2			158.98	

The results of the XPS survey spectrum (Fig. 7a) indicated that C (67.74%), N (4.68%) and O (26.39%) were the main elements of the *S. marcescens* cell surface before adsorption of Y(III). A yttrium peak was identified in the spectrum of *S. marcescens* biosorption of Y(III). Additionally, the AC% of C, N, O and Y were about 53.56%, 8.72%, 33.26 and 4.47%, respectively. This result confirmed that the yttrium was adsorbed onto the cell surface. A decrease in the total C and an increase in total N and O were resulted from the involvement of these elemental atom groups during biosorption^48^.

Two peaks at 157.23 eV and 159.28 eV (Fig. 7b and Table 5) were allocated to the Y3d_5/2–3/2_ doublet in the Y-OH group, accounting for 72.85 AC%^49^. Moreover, the ratio of Y3d_5/2_ and Y3d_3/2_ was 1.497, which was the same as the theoretical value of 1.5^50^. These results indicated that the –OH group involved in biosorption. Two peaks with low intensities (27.15%) and high BE were identified at 158.98 eV and 161.08 eV, respectively. This result reflecting the Y3d_5/2–3/2_ doublet of the yttrium hydroxycarboxylate from the binding of yttrium with carboxyl^50,51^. Furthermore, no redox reactions occurred during biosorption due to the characterized peak of element yttrium at 156 eV was not appeared in the spectrum of Y3d^49,50^. The analysis of the Y3d XPS confirmed that the hydroxyl and carboxyl groups involved in biosorption, and no redox reactions occurred in the biosorption.

Five peaks appeared in the C1s spectra of *S. marcescens* before biosorption of Y(III) (Fig. 7c). These peaks were allocated to the C atom in C–H/C–C, C–O, O-C=O, C=O, O–C–O, respectively. These groups mainly derived from sugars, alcohols, polysaccharides and proteins of *S. marcescens*^52,53^. In comparison with *S. marcescens* before biosorption of Y(III)*,* BE of C=O and COO^−^ recorded a negative shifts to 288.8 eV and 290.31 eV, and the shift was 0.5 and 1.99 eV, respectively. In comparison, C–O and O–C=O peaks recorded a positive shifts to 286.2 eV and 287.6 eV, respectively. The shift of these peaks indicated that the involvement of these carbon groups in the Y(III) biosorption. The shift of the BE of C=O and COO^−^ to lower frequencies after biosorption of Y(III) indicated a decrease of electron density at the adjacent carbon atoms resulted from the donated of electrons oxygen atoms to Y(III)^54^. The replacement of H^+^ by Y (III) should be responsible for the positive shift of C–O and O–C=O^45^. The results of C1s confirmed the involvement of (C–O) and COO^−^ in the biosorption of Y(III).

Two peaks with BE of 399.8 eV and 401.6 eV were observed in the N 1 s spectra of *S. marcescens* before biosorption of Y(III) (Fig. 7e and Table 5). These peaks were allocated to N–C=O/NH_2_ which derived from the amino acid groups of proteins and oxidation states of N atoms with positively charged R-NH_3_^+^, respectively^43^. Apart from the negative shift of N–C=O/NH_2_ and R-NH_3_^+^, a new peak at 403.3 eV was observed in the spectra of *S. marcescens* biosorption Y(III) (Fig. 5f and Table 5). The negative shift of R-NH_2_ and R-NH_3_^+^ peaks could be associated to the variations in nitrogen atom electron density by the formation of a covalent bond between Y(III) and N^54^. The peak at 403.3 eV can be assigned to R-NH_2_Y(III). The results confirmed that the involvement of N–C=O/NH_2_ and most likely complexation action occurred during Y(III) biosorption.

Figure 7g and Table 5 show the assignments of the O 1s spectra. Peaks appeared at 531.5 eV, 532.9 eV and 534.82 eV corresponded to carbon atoms in the forms of –OH/Y–OH, C=O/C–O and COO^−^, respectively. These groups were derived from the sugars, amino acids and ether, respectively^18,44^. The formation of yttrium hydroxide was identified by a negative shifts of –OH from 531.5 to 531.0 eV, and an increase in AC% from 17.09% to 24.8% after biosorption of Y(III)^50^. The peaks at 532.9 eV and 534.82 eV (carboxyl and ether) were shifted to 533 eV and 534.7 eV after biosorption (Fig. 5h), respectively. These were possibly resulted from the variation of electron density for the binding of C–O and C=O with Y(III) via complexation^43^. The formation of a yttrium complex on the *S. marcescens* surface was identified based on an increase in the AC% of total oxygen according to Ramrakhiani et al.^48^. These results confirmed the involvement of C–O, OH and C=O groups in biosorption.

The biosorption mechanism of *S. marcescens* for Y(III) can be proposed based on the above results. Firstly, Y(III) ions were attracted to the surface of *S. marcescens* via electrostatic attraction due to *S. marcescens* cells are negative charged when the solution pH exceeded 4.47. The adsorption was weak because it belongs to physic sorption phenomena^18,55^. Then, the Y(III) ions adjacent to the cell surface could prefer to exchange with Na^+^, K^+^, Mg^2+^and the other light metal ions on the surface of *S. marcescens* through ion exchange, and the biosorption is stronger than that of electrostatic attraction as it belongs to chemical sorption. Finally, Y(III) ions favored of react with the adsorption functional groups (–NH_2_, –OH and –COOH) on the *S. marcescens* surface to form stable complexes than the other elementary mechanisms^18^.

## Conclusion

Single factor experiments showed that pH, initial Y(III) concentration, biosorbent dosage, and stirring speed were the important biosorption parameters of *S. marcescens* for Y(III). The maximum adsorption capacity reached 123.65 mg/g at the optimal conditions of pH 5.5, stirring speed of 169 rpm, Y(III) concentration of 56.41 mg/L, biosorbent dosage of 0.33 g/L, and biosorption temperature of 25 °C for 240 min. The biosorption performance showed that the *S. marcescens* had a good adsorption capacity for Y(III). The characterization of FETEM revealed that the Y(III) ions were adsorbed onto the outer surface of *S. marcescens* cells. Distinct biosorption performance at different pH confirmed that the occurrence of electrostatic attraction in the biosorption. SEM–EDS characterization confirmed the Y (III) ions were exchanged with K^+^, Na^+^, Mg^2+^ and other light metal ions on the surface of *S. marcescens*, which confirmed the contribution of ion exchange to the biosorption. Furthermore, the FTIR and XPS characterization confirmed that the *S. marcescens* surface with adsorption functional groups (hydroxyl, carboxyl, and amine) can enhance the biosorption through complexation with Y(III). Therefore, the biosorption Y(III) ions by *S. marcescens* was the combination of three elementary mechanisms including action of ion exchange, electrostatic attraction and complexation. This work provide a promising efficient biosorbent which can be use in the removal of Y(III) ions from wastewater. Moreover, the adsorption capacity of *S. marcescens* can be further improved through enhancement the adsorption functional groups by targeting chemical modification.

## Data Availability

The datasets used and analyzed during the current study are available from the corresponding author on reasonable request.
